# Radiolabeling and Quantitative In Vivo SPECT/CT Imaging Study of Liposomes Using the Novel Iminothiolane-^99m^Tc-Tricarbonyl Complex

**DOI:** 10.1155/2017/4693417

**Published:** 2017-05-31

**Authors:** Zoltán Varga, Imola Cs. Szigyártó, István Gyurkó, Rita Dóczi, Ildikó Horváth, Domokos Máthé, Krisztián Szigeti

**Affiliations:** ^1^Institute of Materials and Environmental Chemistry, Research Centre for Natural Sciences, Hungarian Academy of Sciences, Budapest 1117, Hungary; ^2^Department of Biophysics and Radiation Biology, Semmelweis University, Budapest 1094, Hungary; ^3^Institute of Nuclear Techniques, Budapest University of Technology and Economics, Budapest 1111, Hungary; ^4^CROmed Translational Research Centers, Budapest 1047, Hungary

## Abstract

The in vivo biodistribution of liposomal formulations greatly influences the pharmacokinetics of these novel drugs; therefore the radioisotope labeling of liposomes and the use of nuclear imaging methods for in vivo studies are of great interest. In the present work, a new procedure for the surface labeling of liposomes is presented using the novel ^99m^Tc-tricarbonyl complex. Liposomes mimicking the composition of two FDA approved liposomal drugs were used. In the first step of the labeling, thiol-groups were formed on the surface of the liposomes using Traut's reagent, which were subsequently used to bind ^99m^Tc-tricarbonyl complex to the liposomal surface. The labeling efficiency determined by size exclusion chromatography was 95%, and the stability of the labeled liposomes in bovine serum was found to be 94% over 2 hours. The obtained specific activity was 50 MBq per 1 *μ*mol lipid which falls among the highest values reported for ^99m^Tc labeling of liposomes. Quantitative in vivo SPECT/CT biodistribution studies revealed distinct differences between the labeled liposomes and the free ^99m^Tc-tricarbonyl, which indicates the in vivo stability of the labeling. As the studied liposomes were non-PEGylated, fast clearance from the blood vessels and high uptake in the liver and spleen were observed.

## 1. Introduction

Liposomes represent one of the first novel drug delivery systems that are utilized to improve the therapeutic indexes of various drugs [1, 2]. Liposomes are composed of a phospholipid bilayer, which forms a spherical structure surrounding an aqueous core [3]. Water-soluble compounds can be incorporated into the aqueous compartment of the liposomes, while nonpolar molecules can be trapped in the hydrocarbon chain region of the phospholipid bilayer [4]. The versatility and plasticity of liposomes due to their self-assembled structure make them clinically useful drug carriers, which is demonstrated on the high number of approved liposomal drugs within the nanomedicinal products [5].

Pharmacokinetics and biodistribution of liposomal drugs and in general all nanomedicines are significantly different from that of free drugs; thus their characterization is of key interest [6, 7]. Noninvasive in vivo imaging methods such as positron emission tomography (PET) and single-photon emission computed tomography (SPECT) are frequently used for this purpose [8–13]. Both methods need the labeling of the liposomal carrier with a radionuclide. Similarly to the encapsulation of different drugs into the liposomes, their labeling with radionuclides can be achieved either by encapsulation into the aqueous interior of the liposomes or by conjugation to a lipophilic molecule and incorporation into the phospholipid bilayer.

The most commonly used medical radioisotope is technetium-99m (^99m^Tc) due to its easy accessibility through ^99^Mo/^99m^Tc generator systems and ideal physical properties such as its half-life (6 hours) and gamma energy (140.5 keV). Labeling of liposomes with ^99m^Tc was first reported by surface chelation of the isotope by using stannous chloride as reducing agent [14, 15]. However, the liposomes labeled using this technique were found to be unstable in vivo, similarly to the procedure which utilizes diethylenetriamine-penta-acetic acid (DTPA) either as encapsulated into the aqueous core of the liposomes or as conjugated to the surface of the phospholipid bilayer [16–18]. Entrapment of ^99m^Tc-hexamethyl-propylene amine oxime (HMPAO) complex by encapsulating glutathione in the aqueous core of the liposomes was proposed by Phillips et al., which resulted in stable in vivo labeling for the first time; however the procedure required a purification step after the labeling because its efficiency was in the range of 60% to 85% [19]. One of the most effective labeling instances of liposomes was achieved by using a hydrazine nicotinamide (HYNIC)—lipid conjugate for the surface chelation of ^99m^Tc [20]. The HYNIC labeling of proteins and peptides as well as liposomes is rapid and yields stable radioconjugates and, most importantly, it does not require any postlabeling purification step because its efficiency is usually above 95% [21].

However, there is a need for the continuous development of liposome ^99m^Tc-labeling procedures in order to aid quantitative SPECT imaging [22, 23]. One direction in technetium complex chemistry is the use of novel ligand systems, such as HYNIC, while the other explores the lower oxidation state complexes. ^99m^Tc-tricarbonyl core [24] belongs to this group, in which the oxidation state is +1 in contrast to HYNIC in which the technetium has an oxidation state of +5 [25, 26]. Therefore ^99m^Tc-tricarbonyl core is chemically inert and has a compact spherical shape with small size [24, 27]. As a result of these properties, this complex retains the biological activity of the labeled object. The ^99m^Tc-tricarbonyl has been used for the labeling of a wide range of biomolecules from small tracer molecules to peptides and proteins [28]. Technetium labeling of liposomes using ^99m^Tc-tricarbonyl complex was reported previously [8], but the applied procedure can only be used for liposomes containing a synthetic DTPA-modified phospholipid.

In the present study, a model liposome system composed of phosphatidylcholine (PC) and phosphatidylethanolamine (PE) lipids is chosen to demonstrate the efficiency of the labeling with the novel iminothiolane-^99m^Tc-tricarbonyl complex [29–31]. The studied liposome sample mimics the lipid composition of two clinically approved liposomal products, namely, the Epaxal® and Inflexal V® [32–35]. Liposomal vaccines such as virosomes and archaeosomes represent a subgroup within liposomal drug delivery systems. Liposomal formulations have many advantages over classical vaccines as discussed in a recent review by Schwendener [36]. The proposed labeling procedure can be directly used to label such liposomal formulations, but its application goes beyond that subgroup. Possibilities concerning the use of the proposed labeling procedure for the quantitative characterization of the in vivo biodistribution of other drug delivery systems are also discussed in this paper.

## 2. Materials and Methods

### 2.1. Liposome Preparation and Characterization

Synthetic high purity 1,2-dioleoyl-*sn*-glycero-3-phosphocholine (DOPC) and 1,2-dioleoyl-*sn*-glycero-3-phosphoethanolamine (DOPE) were purchased from NOF Corporation (Japan). All chemicals were used without further purification. Unilamellar liposome samples were prepared by the hydration, freeze-thaw, and extrusion method. Briefly, the components in the weight ratio of DOPC : DOPE = 4 : 1 were dissolved in chloroform. The solvent was then evaporated at 40°C and the resulting lipid film was kept in vacuum overnight to remove residual traces of the solvent. 10 mM, pH = 7.4 phosphate buffered saline (PBS, Sigma-Aldrich, Hungary) buffer solution made of ultrapure water (18.2 MΩcm) was added to the samples to gain a lipid concentration of 10 mg/ml. Ten freeze-thaw cycles by using liquid nitrogen and lukewarm water bath were applied for homogenization. Finally, the samples were extruded (at 60°C) ten times through polycarbonate filters (Nuclepore, Whatman Inc.) with 100 nm pore size using a LIPEX extruder (Northern Lipids Inc., Canada).

The size distribution of the liposomes was characterized by dynamic light scattering (DLS). DLS measurements were performed using a W130i apparatus (Avid Nano Ltd., High Wycombe, UK) and using a low volume disposable cuvette (UVette, Eppendorf Austria GmbH). Morphology of the studied liposomes was investigated by freeze-fracture transmission electron microscopy (FF-TEM). Fracturing of 2 *µ*L sample was performed at −100°C in Balzers freeze-fracture device (Balzers BAF 400D, Balzers AG, Vaduz, Liechtenstein). The replicas of the fractured faces etched at −100°C were made by platinum-carbon shadowing and then cleaned with a water solution of surfactant and washed with distilled water. The replicas were placed on 200-mesh copper grids and examined in a MORGAGNI 268D (FEI, Netherlands) transmission electron microscope.

### 2.2. Surface Modification with 2-Iminothiolane and ^99m^Tc-(CO)_3_ Labeling

**Figure 1:**
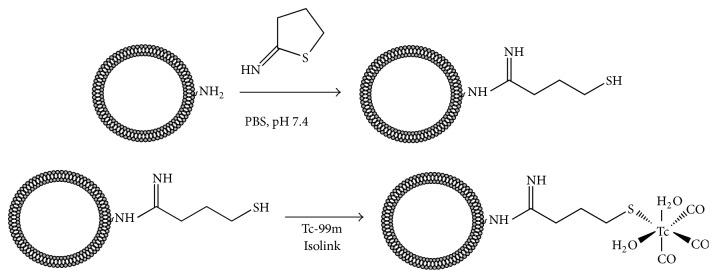
Surface modification of liposomes with 2-Iminothiolane (Traut's reagent) and labeling with ^99m^Tc-tricarbonyl complex.

The surface modification and radiolabeling of liposomes were performed as shown schematically in Figure 1. The prepared liposome sample was derivatized through the primary amine group of DOPE. The free amino group of DOPE was converted into mercaptobutyrimidyl group using 2-iminothiolane (2-IT or Traut's reagent, Sigma-Aldrich, Hungary) [29–31]. 900 *µ*L of liposome suspension containing 2.42 *μ*mol of DOPE was mixed with 100 *µ*L stock solution of 2-IT (14 mM solution in PBS) corresponding to 1.4 *µ*mol of 2-IT. The solution was incubated for one hour at room temperature. The reaction mixture was purified with a PD-10 desalting column (GE Healthcare Life Sciences) collecting 1 mL fraction. The concentration of the thiol-groups in each fraction was determined by using Ellman's reagent (5,5′-dithio-bis-(2-nitrobenzoic acid), DTNB). 50 *µ*L from each fraction was mixed with 50 *µ*L DTNB solution (2 mM in 50 mM sodium-acetate) and with 900 *µ*L Tris buffer (0.1 M at pH = 8). The absorbance values were read at 412 nm using an UV-Vis spectrophotometer (Hewlett-Packard Agilent 8453), and a calibration curve was produced using L-cysteine (Alfa Aesar) solutions ranging from 5 to 30 *µ*M concentration.

The liposome sample with thiol-surface modification was radiolabeled with ^99m^Tc-tricarbonyl complex [^99m^Tc(CO)_3_(H_2_O)_3_]^+^ using a commercial kit (Isolink®, Mallinckrodt Medical B.V.), according to the manufacturer's instructions. 0.5–1.5 GBq [^99m^TcO_4_]^−^ was eluted in 1 mL saline and added to the kit, followed by placing the vial into boiling water for 20 minutes. The alkaline solution was neutralized by the addition of approx. 200 *µ*L 1 M HCl solution. 1 mL of the surface modified liposome solution was added to the solution of the ^99m^Tc-tricarbonyl complex and incubated for one hour at room temperature.

Radiochemical purity was determined by size exclusion chromatography (SEC) using two different approaches. In the first set-up, a PD-10 gravity column (GE Healthcare Life Sciences) was used, in which the chromatography resin is Sephadex G-25 that is able to separate molecules larger than 5 kDa from small molecules and ions, such as the free Tc-tricarbonyl complex. The column was eluted with PBS pH 7.4 and 1.5 mL fractions were collected and their activities were subsequently measured with a Dose Calibrator ISOMED 2010 (MED Nuklear-Medizintechnik Dresden GmbH, Germany) equipment.

Additionally, SEC measurements were performed with a Jasco HPLC system (Jasco, Tokyo, Japan) consisting of a PU-2089 pump with an UV-2075 UV/Vis detector, a Gamma-RAM radioactive detector (LabLogic Systems Inc.) and a Rheodyne 7725i injector controlled by the Chromnav software v. 1.17.02. Tricorn 5/50 high performance column filled with Sepharose CL-6B gel (GE Healthcare Life Sciences) was used for the separation of the radiolabeled liposomes. The fractionation range of this chromatography resin is from 10 kDa to 4000 kDa for globular proteins, which is not only applicable to separate the liposomes from small molecular compounds, but also can be used for the separation of serum proteins from the liposomes. This column was used for the quantitative determination of the radiochemical purity of the labeled liposomes, as well as their in vitro stability in fetal bovine serum (FCS, Sigma-Aldrich). For the stability measurements, 100 *µ*L of labeled liposome sample was mixed with 900 *µ*L of FCS, and 20 *µ*L of the mixture was injected into the HPLC system at time points of 0, 30, 60, 90, and 120 minutes after mixing. PBS pH 7.4 was used as eluent in all HPLC investigations.

### 2.3. In Vivo SPECT/CT Imaging

In vivo imaging was carried out on BALB/c mice (*n* = 3, Charles River Hungary). The body mass of the experimental animals was 28 ± 5 grams and they were 10–12 weeks old. Animal experiments were carried out at the Nanobiotechnology & In Vivo Imaging Center of the Semmelweis University, with permission from the local institutional animal ethics committee number XIV-I-001/29-7/2012 and in compliance with the relevant European Union and Hungarian regulations. Images were acquired with a NanoSPECT/CT Silver Upgrade (Mediso Ltd., Hungary) sequential animal SPECT/CT imaging system. In the SPECT/CT experiment 50 ± 10 MBq of ^99m^Tc-labeled liposomes in 200 *µ*l volume was injected into the tail vein of the mice. Control measurements (*n* = 3) were performed by injecting ^99m^Tc-tricarbonyl complex only. During the scans animals were constantly anaesthetized using a mixture of 1–1.5% isoflurane and medical oxygen. The CT and subsequent SPECT imaging lasted 10.5 and 30 min, respectively. The body temperature of the animals was maintained at 37°C throughout the scanning.

The reconstructed voxel size was 300 *µ*m in 120 × 120 × 328 pixels' matrix both in SPECT and in CT modalities. Reconstructed, reoriented, and coregistered images were further analyzed with Fusion (Mediso Ltd., Hungary) and VivoQuant (inviCRO LLC, US) dedicated image analysis software by placing appropriate volume of interests (VOIs) on the organs. The VOIs were delineated manually on each of the CT scans. Radioactivity concentrations in MBq/cm^3^ were determined for each VOI and corrected for scattering and isotopic decay in the reconstruction algorithm. The uptake values were measured in the following organs: heart, lungs, brain, liver, kidneys, spleen, bladder, and vascular system. The data were evaluated 18, 48, 78, and 108 minutes after the administration of the control ^99m^Tc-tricarbonyl complex and 43 and 92 minutes after the administration of the radiolabeled liposomes.

## 3. Results and Discussion

**Figure 2:**
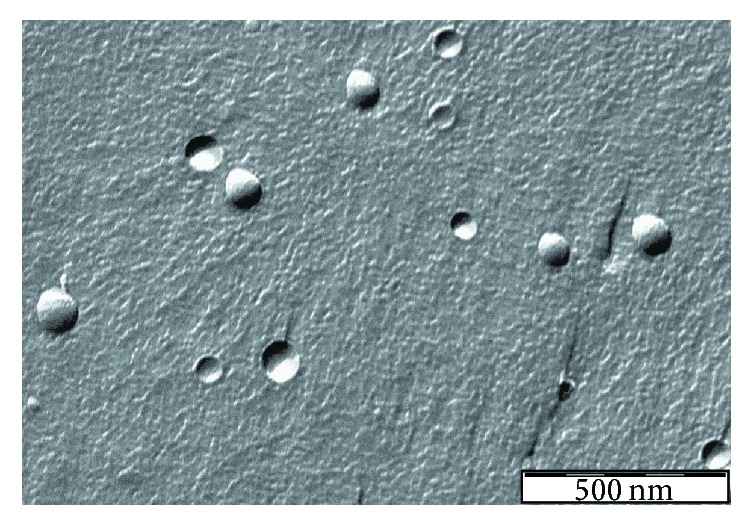
The morphology of the labeled DOPE/DOPC liposomes mimicking the commercial virosome formulations as revealed by FF-TEM.

The model liposome system which was used to demonstrate the proposed new technetium labeling via the novel iminothiolane-^99m^Tc-tricarbonyl complex mimics the lipid composition of two clinically approved liposomal formulations, which contains DOPE and DOPC (20 : 80; wt/wt). Besides those phospholipids the virosomal formulations also contain hemagglutinin, neuraminidase, and phospholipids from influenza virus. Since the presented labeling procedure utilizes the primary amino groups of the phosphatidylethanolamine, we used DOPE and DOPC containing liposomes to model the virosomes.  Figure 2 shows the TEM picture of the freeze-fractured replica of the studied sample. Fracturing takes place between the two leaflets of the phospholipid bilayer, because the van der Waals forces between the monolayers are weaker than the hydrogen bonds between the phospholipid headgroups and the surrounding water. Therefore, the fractured surface shadowed by the platinum evaporation from 45 degrees shows the traces of liposomes broken out from the surface or the traces of liposomes with one monolayer peeled off during the fracturing. FF-TEM revealed homogeneous spherical liposomes in the size range of 100 nm. The average hydrodynamic size of the liposomes according to the DLS measurement is 110 nm with a polydispersity index of 0.05 which corresponds to 22.5 nm standard deviation of the size distribution. In summary, the FF-TEM and DLS results reveal a monodisperse and homogeneous liposome system, which mimics the Epaxal and Inflexal V virosomes regarding their morphology and lipid composition.

**Figure 3:**
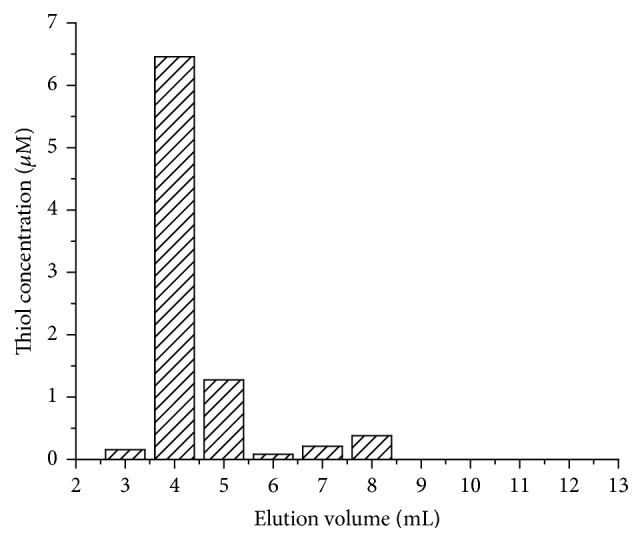
The thiol concentration determined by Ellman's method of each fraction of the elution of the DOPE/DOPC liposome sample after surface modification by Traut's reagent.

The first step of the labeling is the thiol-surface modification of the liposomes, which was achieved by the use of Traut's reagent as shown in Figure 1. The unsaturated bond of the reagent reacts with the primary amino group of the DOPE lipids and forms a free thiol-group via a ring-opening reaction. The reaction mixture was fractionated on a PD-10 column and the thiol concentration in each fraction was determined by the use of Ellman's reagent (Figure 3). The stationary phase of the PD-10 column is Sephadex G-25, which has a fractionation range of 1–5 kDa; hence liposomes are excluded from the gel, while small molecules are eluted at later fractions corresponding to the total volume of the column. Free thiols react with Ellman's reagent in a stoichiometric manner, and the resulting 2-nitro-5-thiobenzoate dianion gives a yellow color which can be used for the determination of the thiol concentration by measuring the absorbance at 412 nm. Figure 3 shows the thiol concentration of each fraction from the elution of the reaction mixture on the PD-10 column. Taking into account the concentration of DOPE lipids in the sample (2.4 *μ*mol in 1 mL), the result presented in Figure 3 indicates that the first two fractions (at 4 and 5 mL elution volumes) in which the liposome is eluted contain 0.0077 *μ*mol thiol-group, which means that 3.2 mol% of the DOPE lipids is modified. Considering that the DOPE represents 20 wt% of the total lipids, the applied surface modification affects only the minority of the constituents, which ensure the validity of the assumption that the biological effect of the labeling is negligible.

**Figure 4:**
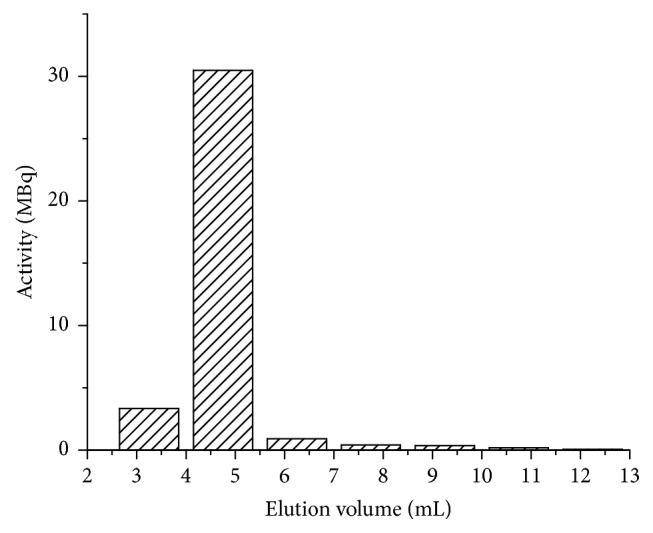
Elution profile of the ^99m^Tc-labeled liposomes on a PD-10 column. The first two fractions contain the labeled liposomes, while the free ^99m^Tc-tricarbonyl is eluted with the total volume of the column.

After the surface modification of the liposomes with Traut's reagent, the free thiol-groups were used to label the surface of the liposomes with the ^99m^Tc-tricarbonyl complex as described in the Materials and Methods. The crude reaction product was separated using a PD-10 column and the activity of each fraction was measured separately (Figure 4). In parallel, the sample was also analyzed by radio-HPLC-SEC using a Sepharose CL-6B gel (the corresponding chromatogram can be found in the Supplementary Information, in Supplementary Material available online at https://doi.org/10.1155/2017/4693417). The labeling efficiency according to the PD-10 column was found to be in the range of 90 to 95% which was also confirmed by the radio-HPLC-SEC measurement. The achieved specific activity—which is a crucial parameter regarding the quantification of the SPECT imaging results—was 50 to 55 MBq per 1 *μ*mol total phospholipid.

The stability of the labeling in serum was also assessed by radio-HPLC-SEC using the Sepharose CL-6B gel, which separates the liposomes from the serum proteins and small molecules. Only 6% of the total radioactivity became detached from the liposomes after 2 hours' incubation in serum (data shown in the Supplementary Information), which corresponds to a good in vitro stability.

**Figure 5:**
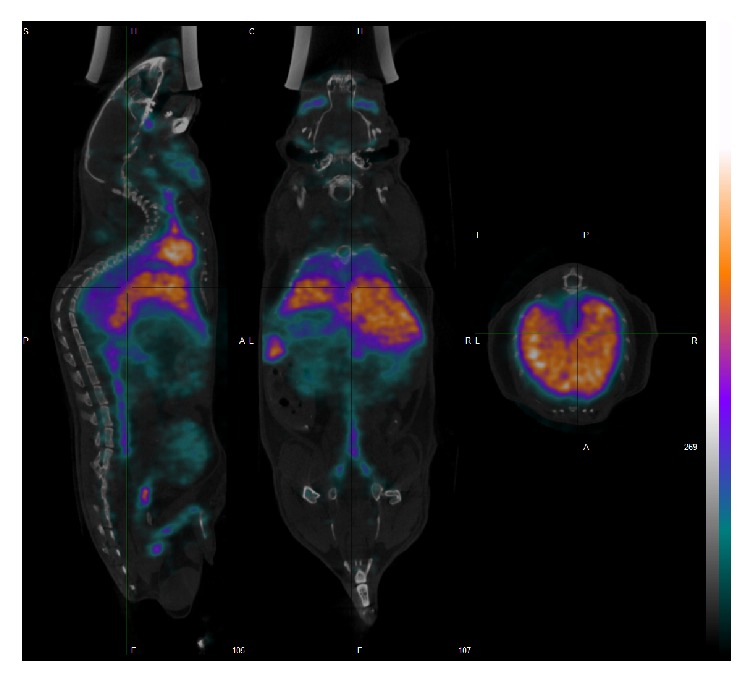
In vivo SPECT/CT images of the ^99m^Tc-labeled liposomes. The 3D reconstructed and coregistered SPECT and CT image are shown together with sagittal, coronal, and axial images (from left to right). Uptake of the liposomes by the liver and spleen is clearly visible in the images.

In vivo SPECT/CT investigations were performed using the labeled liposomes and with the free ^99m^Tc-tricarbonyl as control. Appropriate VOIs were used to derive the activity in the specific organ.  Figure 5 shows a typical SPECT/CT image of the distribution of the labeled liposomes at 92 minutes after administration. High uptake of the labeled liposomes in the liver, spleen, heart, and vascular system can be observed in accordance with the reported distribution of non-PEGylated liposomal systems.

**Figure 6:**
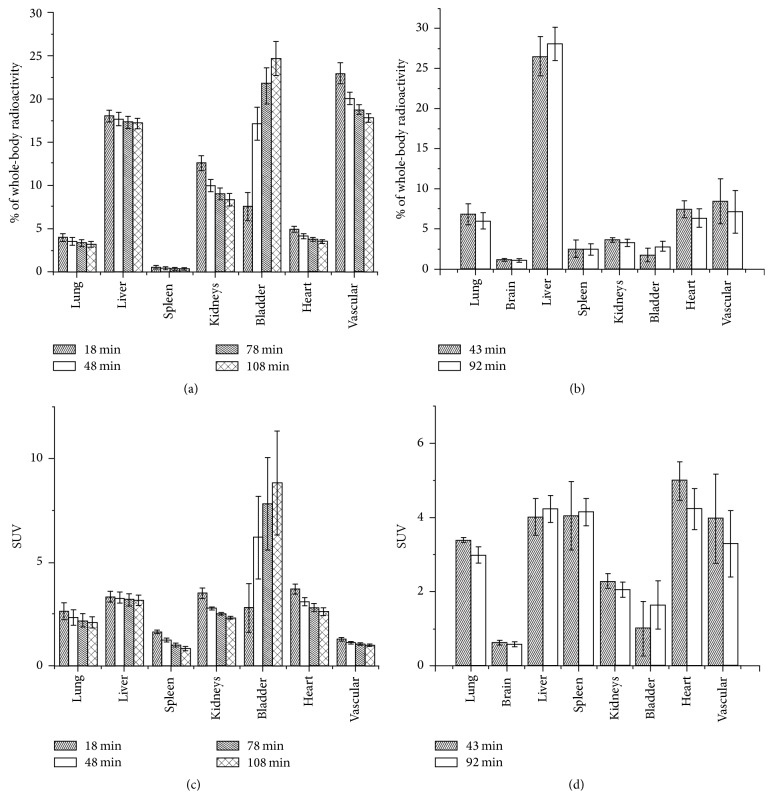
In vivo distribution of the free ^99m^Tc-tricarbonyl complex (a and c) and that of the ^99m^Tc-labeled liposomes (b and d) represented in the percentage of the whole-body radioactivity and as the standardized uptake values (SUVs) for the specified organs. Values were calculated based on the volume of interests (VOIs) corresponding to the different organs and include the radioactivity of the tissue and the blood pool of the organ.

Figure 6 summarizes the distribution of the free ^99m^Tc-tricarbonyl (Figures 6(a) and 6(c)) and that of the labeled liposomes within the different organs as a percentage of the total injected radioactivity and also as standardized uptake value, known as SUV (Figures 6(b) and 6(d)). SUV is defined as the ratio of the tissue radioactivity concentration and the injected activity divided by the body weight (as often used in PET). It should be noted that the in vivo distribution values are calculated based on VOIs and include the radioactivity of the tissue and the blood pool of the organ; therefore it gives an estimate on the organ uptake. Comparison of the distribution of the liposomes and that of the free ^99m^Tc-tricarbonyl shows significant differences. Free ^99m^Tc-tricarbonyl is being gradually cleared from the circulation during the investigated time frame, which is reflected in the decreasing radioactivity in the kidneys and the vascular system and increasing radioactivity in the bladder reaching 24.6 ± 1.96% of the whole-body radioactivity at 108 min p.i. In contrast, the distributions of the labeled liposomes recorded at the two different time points show no significant difference. In case of the liposomal sample, only 1.7 ± 0.82% and 2.8 ± 0.59% of the whole-body radioactivity are detected in the bladder at 43 min and 92 min p.i., respectively, which indicates a good in vivo stability of the labeling.

The aim of the current manuscript is to introduce the developed novel liposome labeling procedure, and the detailed analysis of the obtained distribution is out of the scope of the paper. However, the comparison of the obtained results with previous studies on the distribution of liposomes indicates that the findings reported in this paper show the expected behavior of non-PEGylated liposomes, that is, considerable accumulation in the liver and spleen. On the other hand, the reported in vivo values may be different from ex vivo values in the heart, liver, and kidneys, because of the contribution from the blood pool of these organs.

It is worth pointing out that most of the studies deal with the biodistribution of PEGylated liposomes, and, to the best of our knowledge, the radiolabeling and in vivo nuclear imaging study of liposomes mimicking the lipid composition of Epaxal and Inflexal V has not yet been reported in the literature. The proposed labeling procedure utilizes the primary amino group of the PE lipids, which is the constituent of the used liposomal formulation, but most of the formulations contain only PC lipids, PEG-lipids, and cholesterol. However, conventional liposomal formulation can also be labeled with the proposed method after insertion of PE lipids, amine-functionalized PEG-lipids, or lipid derivatives with thiol-groups via the micellar fusion or the detergent dialysis methods used for the reconstitution of amphiphilic compounds into preformed liposomes [37, 38]. In a wider sense, the introduced method can be utilized to any drug delivery system with amino groups on their surface and results in a stable labeling with high specific activity.

## 4. Conclusions

A novel procedure is presented in this paper for the surface labeling of liposomes with ^99m^Tc by using the iminothiolane-^99m^Tc-tricarbonyl complex. The proposed method utilizes the primary amino groups of the PE lipids and uses Traut's reagent for the formation of thiol-groups on the liposomal surface. The surface modification was verified using Ellman's test, which revealed that only the minority of the PE lipids is modified, which ensures that the labeling does not alter significantly the in vivo behavior of the liposomes. The specific activity achievable for the ^99m^Tc labeling of liposomes with the presented method is among the highest reported in the literature, which enables the quantitative SPECT imaging. The small size and low oxidation state of ^99m^Tc in the complex ensure the good in vitro and in vivo stability of the labeling. The presented method is applicable to other liposomal formulations as well after the post insertion of PE lipids into the liposomes but also to other drug delivery systems, which either contain primary amino groups (such as poly(amidoamine) dendrimers) or can be aminated (such as silica nanoparticles).

## Supplementary Material

The supplementary information contains "HPLC chromatograms".
