# Supplementary material for: Radiolabeling and Quantitative In Vivo SPECT/CT Imaging Study of Liposomes Using the Novel Iminothiolane-99mTc-Tricarbonyl Complex
Source: Contrast Media Mol Imaging. 2017 May 31;2017:4693417. doi: 10.1155/2017/4693417 (PMC5612672; doi:10.1155/2017/4693417)

**Supplementary Information**

**Radiolabeling and quantitative in-vivo SPECT/CT imaging study of liposomes using the novel iminothiolane-^99m^Tc-tricarbonyl complex**

Zoltán Varga^1*^, Imola Cs. Szigyártó^1^, István Gyurkó^1,2^, Rita Dóczi^2^, Ildikó Horváth^3^, Domokos Máthé^3,4^, Krisztián Szigeti^3^

^1^ Institute of Materials and Environmental Chemistry, Research Centre for Natural Sciences, Hungarian Academy of Sciences, H-1117 Budapest, Hungary

^2^ Institute of Nuclear Techniques, Budapest University of Technology and Economics, H-1111 Budapest, Hungary

^3^ Department of Biophysics and Radiation Biology, Semmelweis University, H-1094 Budapest, Hungary

^4^ CROmed Translational Research Centers, H-1047 Budapest, Hungary

*Corresponding author. E-mail address: [varga.zoltan@ttk.mta.hu](mailto:varga.zoltan@ttk.mta.hu) (Z. Varga).

1. The radio-chromatogram of the liposomes labeled with iminothiolane-^99m^Tc-tricarbonyl complex measured with HPLC-SEC using a Tricorn 5/50 glass column filled with Sepharose CL-6B gel.


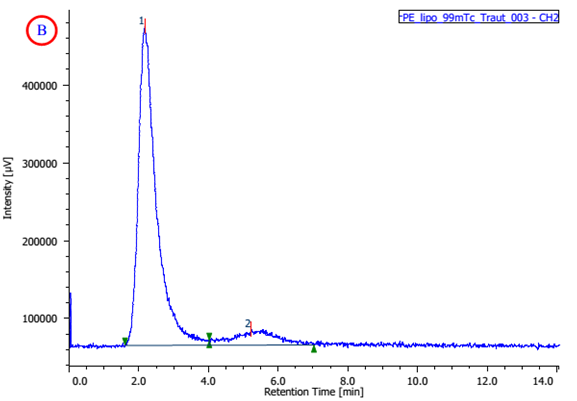


2. The radio-chromatogram of the liposomes labeled with iminothiolane-^99m^Tc-tricarbonyl complex purified on a PD-10 column and measured with HPLC-SEC using a Tricorn 5/50 glass column filled with Sepharose CL-6B gel.


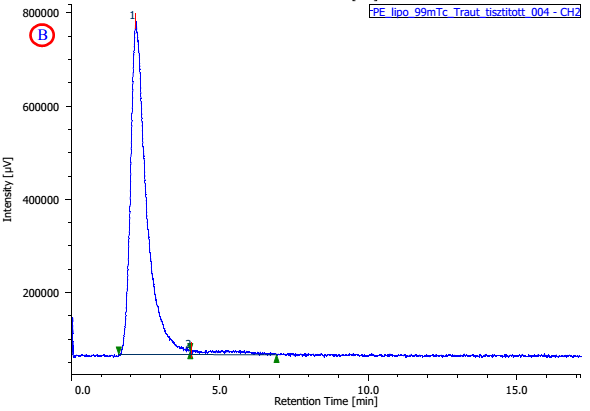


3. The UV (A) and the radio-chromatograms (B) of the labeled liposomes incubated in 90% fetal calf serum for 2 hours. The peak no 1. corresponds to the liposomes, no. 2 to larger plasma proteins up to 4000 kDa, while peak no. 3 to serum albumin and smaller plasma proteins. Only minor fraction of the radioactivity is detached from the liposomes according to the radio-chromatograms.


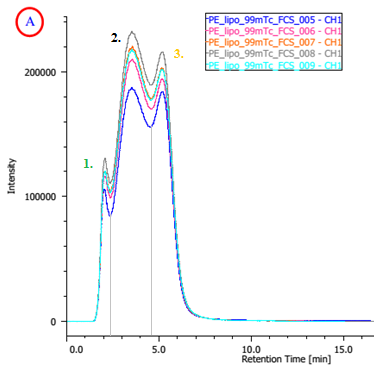

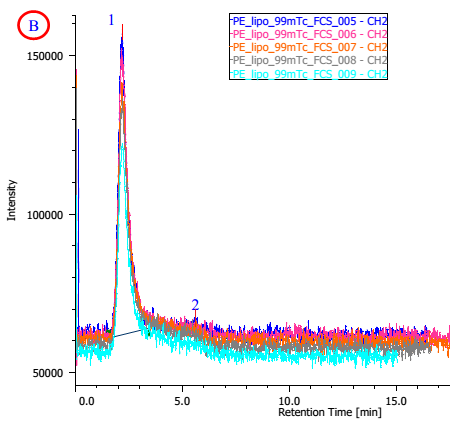

Supplement: Supplementary file 1 — The supplementary information contains "HPLC chromatograms". [file 4693417.f1.docx]
